# Fostering Engagement, Reflexivity, and 21st-Century Skills in Middle School: A Pilot Collaborative Action Research on Identity Formation with Adolescent Co-Researchers

**DOI:** 10.3390/jintelligence10030064

**Published:** 2022-09-06

**Authors:** Pascale Haag, Titouan Fantoni, Stéphanie Dubal

**Affiliations:** 1 École des Hautes Études en Sciences Sociales (EHESS), 75006 Paris, France; 2 Laboratoire BONHEURS, CY Paris Cergy University, 95011 Cergy-Pontoise, France; 3 Institut du Cerveau—Paris Brain Institute—ICM, Sorbonne Université, Inserm, CNRS, 75013 Paris, France

**Keywords:** adolescence, social and emotional skills, identity, children co-researchers, action research, gender

## Abstract

Identity construction during adolescence constitutes a primary psychosocial developmental task. A growing body of research has addressed the importance of school education in fostering adolescents’ identity formation and the skills they need to thrive. Although several studies aimed at defining the factors contributing to a coherent, stable, and integrated identity formation, none sought to investigate this question from the adolescents’ perspective. This contribution aimed to explore new ways of fostering 21st-century skills among adolescents through action research. Five adolescents aged 13 to 15 participated in the research process, creating a survey to answer a research problem mainly focused on identity construction in adolescence. A reflexive analysis of the co-research process highlighted the interest in involving adolescents as co-researchers to foster their social and emotional skills. The deployment of the resulting survey in a sample of 1210 adolescents from the general population highlighted the importance of gender diversity for constructing various dimensions of identity.

## 1. Introduction

How education can foster healthy identity development among adolescents and the acquisition of transversal skills—collaborative, social, emotional, and civic skills—to enable them to face the challenges of the 21st century is a challenging question for educators, as well as for parents and researchers. Identity construction during adolescence constitutes a primary psychosocial developmental task (Branje 2022; Erikson 1968; Helve 2019; Negru-Subtirica et al. 2017). This process involves a “complex interplay of intrapsychic processes and interpersonal experiences” (Abbasi 2016). The transition from childhood to adulthood is rendered increasingly difficult in a rapidly changing world, where new challenges, such as globalization, development of technologies, increasing individualism, and climate change are likely to influence the development of adolescents, their social relations, and their mental health (Patel et al. 2007). School interventions are needed to foster adolescents’ identity development, support them in developing a positive and coherent sense of self, and help them acquire the skills they need to thrive (Lavy 2020; Tiwari et al. 2020; Verhoeven et al. 2019). Following this lead, we conducted a co-research with adolescents (study 1) that resulted in a second research about essential dimensions of identity (study 2).

### 1.1. Identity Formation during Adolescence

Erikson’s psychosocial development remains an essential theoretical framework for studying identity formation during adolescence (Erikson 1968). He conceives identity as a “fundamental organizing principle which constantly develops throughout the lifespan”. This principle is considered a synthesis of elements from the past (personal history), from the present (needs and personality), and from expectations of the future. This synthesis process is cardinal to adolescent development when one explores various social roles. However, as pointed out by Phillips and Pittman (2007), Erikson’s theory does not lend itself easily to empirical research methods.

Refining Erikson’s work, the identity status paradigm proposed by Marcia (1966, 1980) goes one step further. It is characterized by the adolescent’s levels of identity exploration and commitment to self-chosen goals. Marcia differentiates two processes of identity construction: Exploration—the search for different alternatives for oneself in an area of life—and Commitment—the adhesion to a set of values, aims, and beliefs. Depending on their combinations, four statuses are identified (Marcia 1966; Marcia et al. 1993).

**Figure 1:**
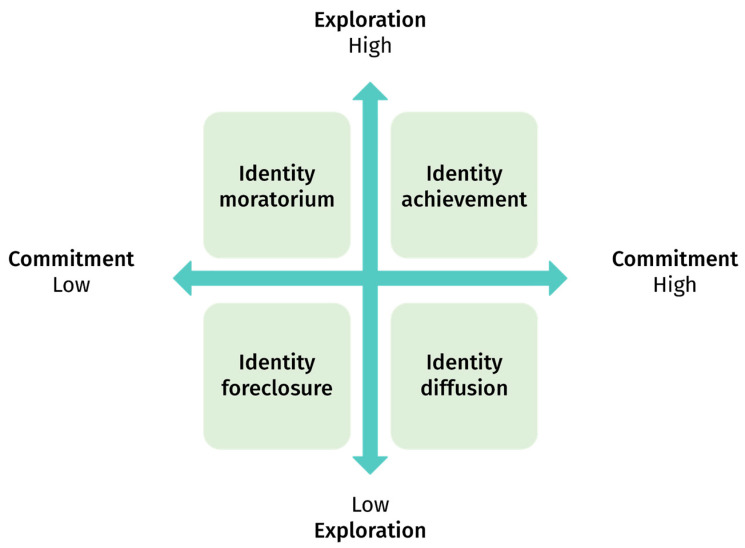
Marcia’s Identity status model (1966).

Figure 1 represents the four statuses’ main characteristics that can be summarized as follows (cf. Marcia et al. 1993, pp. 7–8):

- *Identity achievement*, or *self-constructed* identity, qualifies individuals who tend to build “their own [game plans], not their parents”, seeing “the future as something to be shaped, a period of identity creation or realization rather than a time to meet preset standards”;
- *Identity foreclosure* refers to individuals with *conferred identities* who tend to “adopt a lifelong ‘game plan’ set out for them by their parents or similar authority figures”;
- *Identity moratorium* is used in the case of a “transition from no sense of identity or a conferred to a constructed identity”; individuals are compared to “trapeze performers, holding on to the bar of the past while swinging toward that of the future, often with much of the vacillation, fear, intensity, and excitement connoted by the circus image. At some times, all things seem possible to them; at other times, they can be so totally self-preoccupied that their whole phenomenological world is consumed with their present struggle”;
- *Identity diffusion* or *no firm strong identity* corresponds to the “lack of a coherent identity”, with little “future sense” or “central sense of self”, mostly feeling “subject to the vicissitudes of fortune”, and “whether optimistically or pessimistically, somewhat out of control of their futures”.

Many longitudinal studies found these statuses stable (Meeus 2011). Empirical research found that the statuses with higher engagement and exploration levels show a better psychosocial integration in society, as well as higher levels of well-being and self-confidence and fewer depressive symptoms (Arnold 2017; Meeus 2011) and that the knowledge and understanding of these statuses have solid implications for therapeutic and educational interventions (Kroger and Marcia 2011).

This psychosocial framework of identity development is coherent with Cuin’s (2011) sociological approach to adolescence. For this author, adolescence’s “crisis” would be related to the experimental nature of adolescent behaviors: adolescents tend to move away from previous normative models and test and adopt new ones while privileging those that seem most valuable to them. The adolescents’ ability to manipulate social norms depends on two principles:

1. Integration, which requires the ability to identify, appropriate and subscribe to norms in order to benefit from the psychological and social effects of that subscription.
2. Strategy, which consists in learning to move away from norms that impede access to other types of benefits—either by transgressing them or by cleverly exploiting them.

These two dynamics embody a subjectivation process, i.e., the construction of a social subject, both agent and actor of social norms. Sociology constructs the theoretical framework of adolescence as a moment of autonomy without independence (de Singly 2006), during which the dynamics of integration and strategy allow this subjectivation process. Autonomy refers to identity criteria, whereas independence refers to statutory requirements (Galland 2008). Thanks to social media and other contemporary changes, today’s adolescents have significant decision-making power over their own lives, especially regarding the constitution of their peer groups: they, therefore, have more control over how they fit into social norms (Galland 2008; Metton-Gayon 2006). In this way, sociological and psychological frameworks of identity complement each other, showing how healthy identity development relates to one’s adhesion to social norms.

Finally, Erikson’s psychosocial development theory does not consider that adolescence is the moment identity elaborates as a stable entity for life: identity evolves constantly. This is consistent with Marcia’s model, which considers a person’s identity determination as a process resulting from individual commitments. Such commitments are not made once and for all but can be questioned throughout one’s life.

A healthy process of adolescent formation of identity guarantees a better integration into society. For this, considering well-being and self-confidence in developing identity is essential. Since Erikson’s work, many studies have corroborated that well-being and identity formation are strongly related (Luyckx et al. 2006). The links between well-being and identity styles have also been investigated, indicating a negative association between a diffuse/avoidant style—lack of exploration and commitments, difficulty in setting goals—and various indices of well-being and a positive, hopeful outlook toward the future (Phillips and Pittman 2007). All these studies indicate a positive association between social and emotional skills on the one hand and healthy identity development and well-being on the other.

### 1.2. Fostering 21st-Century Skills among Adolescents

Since the beginning of the millennium, there has been an increased interest in the question of social and emotional skills. They are variously referred to as 21st-century skills, psycho-social skills, non-academic skills, character strengths, soft skills, life skills, or transversal skills (Borghans et al. 2008; Heckman and Kautz 2012). There is no single exhaustive list since different authors worked with other lists. The World Health Organization defines them as “abilities for adaptive and positive behavior that enable humans to deal effectively with the demands and challenges of life” (WHO 1994). It recognizes ten skills grouped into five pairs (problem-solving, decision making, creative thinking, critical thinking, self-awareness, empathy, interpersonal relationship, good communication, management of stress, and management of emotions.)

Several studies have elaborated on this definition and have come to consider these skills as a coherent and interrelated set of psychological abilities involving specific knowledge, intra-psychological processes, and attitudes, which make it possible to increase individuals’ autonomy and empowerment, to maintain a state of psychological well-being, to promote optimal individual functioning and to develop constructive interactions (Kankaras and Suarez-Alvarez 2019; Lamboy et al. 2022; Schoon 2021). In a synthesis compiled for Santé publique France, Lamboy et al. (2022) propose a taxonomy of 22 skills classified under three broad categories—cognitive skills (e.g., awareness, self-control, thinking critically, ability to achieve goals, to make responsible choices or to solve problems creatively); emotional skills (e.g., identifying and understanding emotions and stress, ability to regulate emotions and to manage stress in everyday life, coping skills); social skills (e.g., pro-social attitudes, assertiveness, and constructive conflicts resolution).

A growing body of studies shows their decisive role in the development of mental health, physical health, work performance, and social relations (Mikolajczak et al. 2020). The development of these skills thus represents a significant issue in public health, education, and social action today (Lamboy et al. 2022). School climate and pedagogical practices contribute to the development of a wide variety of skills among pupils and students, such as self-efficacy (Dweck 2016; Usher and Pajares 2008), problem-solving (Baraké et al. 2015), cognitive flexibility, divergent thinking, and creativity (de Vries and Lubart 2019; Scheibling-Sève et al. 2017), social and emotional skills (Oberle and Schonert-Reichl 2017; Osher and Berg 2017). School climate and pedagogical practices can also favor intrinsic or self-determined motivation (Deci and Ryan 2000) as well as prosocial behaviors and civic engagement (Denney 2022).

### 1.3. Fostering Identity Formation: Lack of Interventions

As Schwartz and Petrova (2018) pointed out, the field of identity interventions is still relatively young, and etiological work suggests that interventions may facilitate identity consolidation. Connecting schoolwork with “real-world outcomes” is one of their recommendations to foster adolescent identity development. Incorporating identity development into prevention programs is another avenue of intervention. Moreover, the inherent limitations of interventions designed solely by adults being widely established, relying on peers, promoting adult-youth partnerships to conceive interventions, and placing young people in positions of leadership are likely to help young people develop a healthy and consolidated sense of identity, supported by advocacy and empowerment and leadership (Schwartz and Petrova 2018).

Studies show that a feeling of consistency and coherence within one’s sense of identity is associated with higher levels of well-being and lower levels of depression or anxiety (Meca et al. 2015). By contrast, lack of family and community support, and struggle to integrate various aspects of identity (gender, sexual, religious, cultural, etc.) relate to higher risks of health-compromising behaviors (Schwartz and Petrova 2018). In addition, short-term intervention efforts fail to produce long-term gains (Kroger and Marcia 2011). School support for students’ exploration of their identity is related to civic engagement and positive psychosocial development in adolescence (Crocetti et al. 2014; Kaplan et al. 2014). Finding efficient ways to promote healthy identity development in adolescents is therefore essential.

### 1.4. Children as Co-Researchers

Since the early 2000s, the inclusion of children or young people themselves as co-researchers to better understand their perspective has been the subject of much deliberation, both about the benefits of these new approaches and about their limitations (Bradbury-Jones and Taylor 2015; Camponovo et al. 2021; Lundy et al. 2011; Smith et al. 2002). For young people, participating in a project as co-researchers, thus being involved in the elaboration of a research question, the collection of data and its analysis can contribute to building their self-confidence, improving their critical thinking, autonomy, engagement, and sense of competence (Kellett 2010; Suleiman 2021). In terms of research outcomes, their participation provides more direct access to knowledge derived from children’s own understanding of their environment and subcultures. It, therefore, provides new insights that complement other approaches and enrich the knowledge gained as a result of the research (Bradbury-Jones and Taylor 2015)—having the opportunity to actively contribute to an authentic research project, whether as co-researchers or as joint authors, alongside experienced researchers, also affects adult-adolescent relationships and allows them to make their voices heard rather than being incorporated as a ‘data source’ (Groundwater-Smith and Mockler 2016).

There are, however, also practical and ethical limitations to this approach: on the one hand, children are not trained in research, and it is, therefore, necessary to provide them with some knowledge and skills to participate fully in the project; on the other hand, it is essential to take into account the asymmetric nature of the child- or adolescent-adult relationship, to be aware of the power relationships involved, and to ensure that children’s participation is safe, with their consent, by the ethics of research, and with the possibility of withdrawing from participation at any time (Bradbury-Jones and Taylor 2015; Camponovo et al. 2021; Fielding 2011).

One additional premise of this research was that by involving adolescents in a project which directly resonates with their concerns and by letting them take part in the decision-making, they would gain a deeper understanding of scientific research methods and requirements and establish meaningful relationships with significant others (peers and adults) while sharpening their critical thinking and problem-solving skills (Jacquez et al. 2020; Suleiman 2021). Such practices—i.e., integrating research on topics that are meaningful to the students in the classroom—improve learning-related attitudes, self-efficacy, autonomy, communication skills, teamwork, and collaboration and ultimately lead to increased social support and community transformations (Jacquez et al. 2020).

## 2. Theoretical Framework and Research Question

This paper presents a research process comprising two interconnected studies—the first embedding the second. It aims to explore new ways of fostering 21st-century skills such as critical thinking, collaboration, and sociability among adolescents through a collaborative research project involving up to five adolescent co-researchers (study 1). They have been actively involved at all stages of the co-research process, and the initial discussions led to the design of a questionnaire assessing some cardinal dimensions of identity development from the point of view of adolescents themselves, with an emphasis on gender identity. This questionnaire was used to conduct a survey, the results of which are presented in study 2.

In the framework of collaborative research involving co-researchers of different ages and statuses, the interplay and dynamics emerging at the same time as the work is carried out make it difficult to detect and objectively assess any transformation among the participants who are immersed in the process and are thus not necessarily able to take a step back. Since this project is intended as a pilot study focusing on the effects of being involved in a research project on adolescents’ psychosocial skills, it was necessary to find an appropriate way of assessing the transformations induced by the research setting among its participants.

This led us to resort to Engeström’s *Activity theory*. According to Engeström (1987), one of the limitations of traditional psychological and sociological research lies in the difficulty of understanding change in numerous everyday situations within complex contexts. This observation led him to propose the use of the concept of an Activity System as a unit of analysis (Engeström 2000) and “to understand individual action and support individual and system development, we must study action in the context of the broader activity in which it is taking place” (Daniels and Cole 2002, p. 311). This cross-disciplinary approach is widely recognized as a valuable approach for studying human practices in various fields involving human activity, such as psychology, education, management, culture, and information systems—where individual and social levels are interconnected (Monaghan 2016; Vandebrouck 2018).

**Figure 2:**
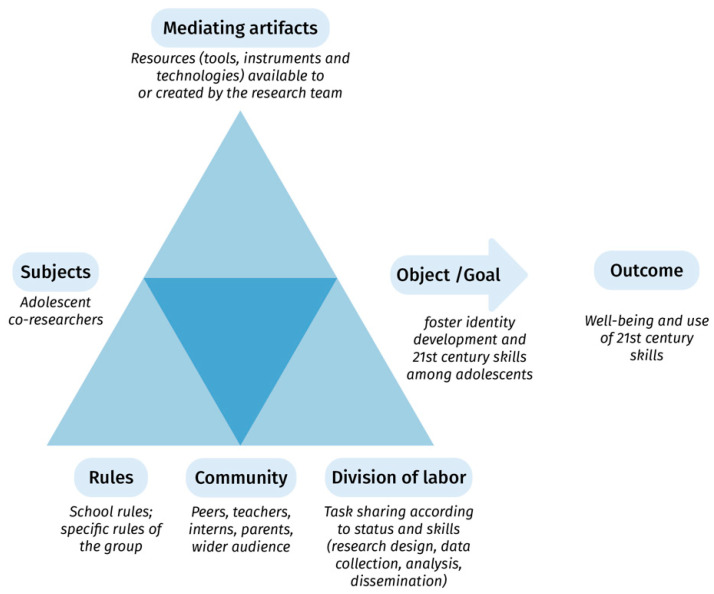
Theoretical framework using Engeström’s Activity theory to analyze the transformation of subjects in this research.

We propose to study the implemented procedure by analyzing it as a “system of activity” instead of focusing on each factor taken in isolation. In the Activity theory framework, an activity or set of activities is considered as mediated by different contextual elements: subject, object, artifacts, etc. The object, sometimes also called the goal, is what motivates the activity. According to Vandebrouck (2018, p. 679), the object is “a characteristic that distinguishes one activity from another”—in our case, “ordinary” classroom activity vs. intentional and systematic attempt to nurture 21st-century skills. The rather heterogeneous category of artifacts also mediates the activity, sometimes also called tools, instruments, or technologies. Artifacts refer to all the resources—already available or created by the subjects—to reach the object; they can be concrete (e.g., digital tools, surveys) or immaterial (e.g., thoughts, decisions, researchers’ skills, feelings). The object leads to an outcome—adolescents’ ability to use such skills. Throughout the process, subjects and objects “form a dialectic unit: subjects transform objects, and at the same time, subjects are transformed” (Vandebrouck 2018, p. 679). For this study, the system is analyzed from the point of view of the adolescent co-researchers. Our activity system can thus be broken down into its component parts and represented as in Figure 2.

The scientific angle chosen for the current project falls within a trend of reflection on the researchers’ posture regarding their research objects and on the place of all the actors involved in a scientific investigation (Camponovo et al. 2020; Lyet 2017). In this context, the usual division of labor between researchers who are “producers of knowledge” and respondents who only possess knowledge of experience and action, excluded from the field of “legitimate knowledge”, is not an option: participants in this project adhere to what some researchers call a new ‘science-society contract,’ which recognizes the role of all actors in the production of knowledge, and for which the key words are ‘participation’ and ‘reflexivity’ (Barré 2017; Bonny 2017). Our approach seeks to overcome the hierarchy of powers and knowledge in line with critical epistemologies. It falls into the broad category of collaborative and partenarial research, which refers to a reflexive partnership aiming at the co-production of ‘actionable’ knowledge, i.e., knowledge built in and for the sake of action (Juan 2021).

More specifically, the authors are entirely in line with the approach known as transformative action research, promulgated by Bilorusky (2021), where research, inquiry, and action are brought together in transformative ways to make a difference. Transformative action research is an organic, evolving process in which action and research affect, influence, and transform each other, acknowledging the use of improvised strategies as part of the process by actively involved actors in the social reality being studied.

This project owes a great deal to that of Camponovo et al. (2020) in the sense that we aim to bring together the points of view of adolescents with those of a research team on a given topic to obtain the most nuanced, comprehensive, and integrated possible perspectives on the knowledge thus produced.

## 3. Study 1: New Ways of Fostering 21st-Century Skills by Involving Adolescents as Co-Researchers

### 3.1. Context and Participants

Lab School Paris is the first French school inspired by the North American model of laboratory schools, pioneered by John Dewey in Chicago at the end of the 19th century. Founded in 2017, it started with 27 pupils aged 8–11. In 2021–2022, around one hundred students from 6 to 15 years old (elementary and middle school levels in the French educational system) were enrolled. Since its opening, Lab School Paris has maintained regular collaborations with a network of researchers linked to various institutions. In particular, since 2019, it has been participating in a European Erasmus+ project entitled LabSchoolsEurope: Participatory Research for Democratic Education that aims to develop and share democratic practices for teaching in heterogeneous classroom settings (Haag 2021).

At the beginning of the school year 2021–2022, some middle school students started discussing gender and sexual orientation issues. For some, these issues caused such discomfort and anxiety that they hindered their learning at school. This was a situation without precedent: since its opening as an elementary school in 2017, the school has been growing along with its students, opening new levels every year. The enrolment of middle school students gave rise to further questions and challenges, such as welcoming adolescents’ concerns or fostering their intellectual and emotional development. In the absence of a predefined framework within the school to enable these questions to be voiced in a safe environment, the founder of the school and first author of this article—a trained psychologist and assistant professor at the École des hautes études en sciences sociales (EHESS, Paris)—proposed to create an ad hoc group for students wishing to participate. This group would be facilitated by an intern from Lab School Paris holding a Master’s degree in philosophy. The idea was to co-construct a framework with the students to express themselves freely without fear of being judged.

Initially, four 8th and 9th grade students, aged 13–15, joined the discussion group named “Gender and Society” for weekly meetings. The initial goal of the meetings was to create a safe space to explore and reflect upon questions related to gender and sexual orientation (this specific topic will be covered in Section 4). The four students involved knew they were welcome to share their thoughts and ideas with their teachers and classmates during weekly student councils. They could also offer suggestions to ensure that all students in the class felt welcome regardless of their gender identity or sexual orientation. The “Gender and Society” group presented their work to the rest of the class^1^.

Neither the direction team nor the teachers participated in these meetings. The fact that the intern was a philosophy graduate with a good knowledge of gender issues may have contributed to creating such a safe space. The second author of this paper, appointed research assistant at the beginning of the action research, is a Master’s student in Gender studies, working on the sociology of gender and education.

In early 2022, the group’s discussions became less active, as if the initial goal had, at least to some extent, been reached—all the students feeling comfortable enough to share their concerns and thoughts with their peers and teachers. The school’s founder then proposed holding a debriefing meeting to reflect upon what they had learned. At this stage, one of the primary outcomes was the students’ realization that (1) gender is one facet of identity but not the only one, and (2) that it is possible to explore these questions without necessarily opting for definitive labels. This could have been the last session. However, a question eventually arose about what could be done to extend this experience to other schools. Informed about research projects involving children or teenagers as co-researchers, the group participants enthusiastically agreed to launch a study. This decision instilled a new dynamic in the working group and marked a significant turning point in the project.

### 3.2. Procedure

The new research group was composed of the four students of the working group, the intern who had facilitated it, a new pupil (age 14) who had joined the school in the meantime, and the first and second author of this paper. During weekly meetings of approximately one hour, the research project was elaborated: definition of a research topic, design, and methodology. Initially, the adolescents were mostly thinking about conducting interviews with other adolescents outside of school to question their perceptions of identity. The main principles of qualitative analysis were briefly explained to them. Once they realized that this approach implied a transcription and analysis of the interviews, they opted for conducting an online survey which was more realistic from a practical point of view, as the end of the school year was nearing.

New questions arose: How do you design a questionnaire? How do you frame the questions? In what order? To what extent can you ask personal questions without risking the participants leaving the survey without completing it (e.g., about gender or sexuality)? All those questions mostly came from the adolescents themselves. During the weekly meetings, they thought about how they would structure the survey and formulate the questions and wondered what would be interesting to ask to collect interesting and relevant data.

Once the questionnaire was ready, we tested a pilot version on a few adolescents outside school. We requested the co-researchers to ask someone they knew to fill in the questionnaire and to give feedback, especially in case something was unclear so that we could make changes to the survey before its dissemination. The corrected questionnaire version was tested among the 26 middle school students at Lab School Paris. After some minor formal changes, the co-researchers decided to conduct a survey using a snowball sampling method: all the co-researchers sent the survey link to as many people as possible; we also sent the survey link to middle schools and high schools found in the French national education Ministry website.

At the end of the school year (June 2022), two meetings were held with the third author of this paper to explain to the co-researchers how to analyze the survey data with statistics; debriefing sessions were also organized so that the students could share their impressions and feedback about the whole research process: what they had learned throughout the process, whether it had changed them and in which way, etc.

Qualitative data –from recordings of the meetings and interviews, as well as notes from the sessions—was analyzed to determine whether participating in this research project had served the purpose of fostering adolescents’ 21st-century skills. The research assistant conducted the interviews during the summer of 2022 in individual zoom meetings or phone calls. The questions followed an interview guide constructed by the first author of this paper. The interviews were relatively short (10–15 min), and we discussed at the end of the interviews which information could be shared publicly in the case that some information was confidential.

Quantitative data from the online survey conducted by the co-researchers in study 1 is presented in Section 4 (study 2).

### 3.3. Results

The qualitative data was examined using the Activity Theory framework: a content analysis was performed on the relevant sections of the interviews and focus groups on understanding how they felt, what they learned, and the kind of change the project had brought up from their point of view. In this section, we only consider the adolescents’ voices, although the whole process has included regular formal and informal sharing of reflections among adults throughout the project.

#### 3.3.1. Artifacts and Division of Labor: Learning and Contributing According to Each One’s Expertise

The students shared their thoughts about what they had learned, which aspects of the project most interested them, and in which ways they had contributed:

*[I] feel like I participated in a little bit of everything too, which is pretty nice; it allows me to have a little bit of experience, see a little bit of the whole process.*

Through the research process, they discovered how to build a survey, participated in its dissemination, and began to get some insight into statistical analysis of the data thus produced:

*I participated in finding out what we were going to do; I also participated in a radio show to disseminate the survey. And I also helped to find the questions for the* survey *[…].*

*[I have acquired] the skills to form a* survey *and the skills of research work.*

*I think [what I participated in the most] was the questions when we wrote them because that’s what we mostly did […] because we weren’t going to do the statistics, obviously, and writing the article is more complicated. We couldn’t do it ourselves, so that’s what we could do the easiest.*

They also recognize how their personal experience would benefit the whole group:

*I think [I contributed with my] perspectives because we all have different views, my experiences from living in New York, I could contribute with that experience, and so by, like, those questions in the survey, I felt like I could contribute with. And also, by being able to share this survey with people I knew all around the world.*

#### 3.3.2. Rules and Social Relationships within the Community

All the participants showed appreciation for the quality of the relationships among the students who participated in the research and in the broader community: they highlighted how much they felt accepted, regardless of their different identities, and how free they felt to express their points of view.

*I felt like I was with people who kind of understood my vision of things. And they didn’t impose their opinion on me, so we had civic conversations about it, which was pretty cool. It’s not just about gender issues; it’s about listening to each other’s opinions instead of saying, “no, you’re wrong, and I’m right”. There was an atmosphere of caring in the group that was quite nice.*

*I experienced an openness to express my own opinions […]. It’s a friendly atmosphere where there can be conflicting views, yet it’s scarce in life in general! There are a lot of opinions, pros, and cons, there are too many opinions, and it messes up everything, everywhere.*

This feedback points out that they felt it was possible to express opinions without being judged. The space of discussion provided by the explicit rules within the research group—confidentiality and absence of judgment—made them feel comfortable. That feeling allowed them to elaborate their thoughts and to gain from others’ perspectives:

*It was a very good environment; it was a very open environment. I feel like we could all really express our positive and negative thoughts. I also felt like I could build my thoughts onto others, and others could build their thoughts onto mine, so we were just helping each other and supporting each other.*

They also felt supported by the adult community in this endeavor:

*I found them [the adults] very open-minded about all these issues […] they were just there to help us put the thing together, because we were kind of the ones formulating the thing, doing the thing, and they were there to guide us, to prevent us from getting overwhelmed. I thought it was pretty cool.*

*I also felt it was nice to have adults in the group to like, guide us and show us how to have a formal survey and guide us into those conversations.*

Not only did the co-researchers benefit from the project, but also, at various stages, we communicated with the rest of the school ecosystem—students, teachers, and parents –whether through discussions with the whole class or the presentation of the first results. This allowed conversations about identity and gender identity within the school with adolescents who were not part of the research group, although some participants expressed their regrets about not having more diversity among the students participating in the research project:

*I would hear other people talking about their relationships with their parents in terms of their sexuality or gender, and I would see how things were going in other families, and that would allow me to see a little bit how I could react to them too. […] Because my parents didn’t talk about it either, until very recently.*

*I think it would have been nice if we had tried not to include but like engage other students more so we could have gotten more into their perspectives. I think it would have been interesting to understand how the project would have affected them, but yeah, it just pushed me to have discussions with other people in the school. […] We were all already pretty close friends, we were all LGBTQ, and I think it would have been beneficial if we’ve had at least like cishet teenagers or just get their perspective, or a person of color as well because we’re all white so, yeah, yeah.*

#### 3.3.3. Reaching the Object: The Point of View of the Subjects

During the final discussions, we explained to the student co-researchers that a critical feature of this project was also to create different relations and collaborations between young people and adults inside the school, to explore new approaches to teaching and learning, and to foster skills and abilities that we considered necessary more than ever in the current context, trusting them with responsibilities and giving them autonomy. We asked them for feedback and how we could improve the process in the future.

*Actually […] you weren’t directing anyone; you were showing paths. […] What I find cool in life is that you can take a word, a text, and there can be thousands of paths […]. And the goal of adults in life, I think, is to support selecting paths and to guide as much as possible.*

*You were super open, super ok to talk about this kind of subject, you listened and everything, you didn’t try to distort, it was cool to talk, we felt that there was no judgment and that we could speak freely and say what we thought and everything. […] There were no questions that implied the answer or were biased. […] I didn’t feel guided or influenced to say answers that weren’t my own.*

Reflexivity and critical thinking emerged while talking about how the setting could be improved:

*Maybe to have a bigger group of adolescents, a slightly more varied group, because we were all very similar in many ways […] and it would have been nice to get a few other teenagers that could add their perspectives, I guess.*

*I think it would be interesting to ask others in the class like what parts of their identities are important ‘cause every single one of us in the group is LGBTQ… [Laughs] Yeah, we need an opinion from a straight person!*

*Maybe not do it at the end of the year, because at the end we were too much in a hurry […] do it at a time when we can help work on it.*

Some of the feedback also indicated that the project allowed the participant to gain more perspective and understanding of who they were:

*It was like, it’s not scary to talk about it, and people are, in fact, nice. Wow. I didn’t know that was possible in school […] And also, I’ve always been super interested in psychology and stuff. And also like social justice, activism, and kind of putting those together, and into a study. I don’t know, it felt like […]. I’ve always had this question What is me? What makes me me? Is it my brain, my consciousness, is it my body, is it … I don’t know … […] I have all those questions. This has begun to answer a few things, organized a few things in my head, and kind of made a start somewhere of what makes us us, what makes us an individual.*

#### 3.3.4. Outcomes: Lessons Learned

The adolescent co-researchers acknowledged that this project helped them to become more aware of their social environment, more reflexive, and more open to others.

(student) *Identities are also a pretty vast subject; it doesn’t stop where we defined it; there’s still a lot more to talk about. […] I always knew that it [identity] was much more than my gender and what I look like, identity; identity is much more than that.*

(adult researcher) *Did it change during this process?*

(student) *Yeah, it kind of expanded.*

They felt that they could share their views on identity more freely, both with their parents/friends and themselves and even publicly, at a conference or in the media. One of them mentioned at the end of the academic year that talking openly within the group allowed him to better identify his feelings as an LGBTQ+ adolescent.

*Until I was 12, I knew the words LGBTQ+, but I didn’t know what they meant. So I couldn’t put what I was feeling into words, so obviously, it was a bit complicated for me, with my parents, and with regard to myself.*

The data they collected helped them learn about themselves through others and had positive effects on their social well-being:

*Something I realized, reasonably major, is that I am not alone. People often say to me, yeah, you’re not the only adolescent asking yourself this kind of question, there are millions of teenagers asking themselves this question, but it’s all very well to talk about it. Still, when you realize that all the people took part in the survey, you say to yourself, “well, yeah, I’m not the only human being on Earth asking myself these kinds of questions”, you feel less alone. There it was concrete; you see the answers of the people.*

Gaining self-confidence through the research project seems partly related to the fact that this study enforced the co-researcher’s ability to pay attention to themselves and others at the same time, without depreciating any of them through comparisons:

*I think [what I learned the most is] diversity. The different stages of development we’re at, just how we all navigate our identities completely differently, even if we are at the exact same age. So just like, looking at the responses, it was just really interesting to see that some people had part of their identities that were way more developed than mine, but other parts that were less developed. It was really interesting to see what parts were the most important.*

Acknowledging individual differences also fostered empathy towards their peers:

*[The aim of such co-research] is not just getting to know ourselves better but to understand others better, to see others’ perspectives. I think that’s really what I gained out of this, other people’s perspectives, and just trying to understand how people do that because I know myself. I know how I do things, and I think it’s really beneficial to gain empathy and compassion to understand someone differently.*

Participating in this project was considered stimulating and made the students proud, as the number of participants in the survey exceeded their expectations:

*Look at that, I made that, all those people, most of them I don’t even know!*

Finally, concrete propositions for new rules inside the school community arose beyond exchanging ideas during the project. Although democratic participation and openness to differences are already part of the Lab School Paris’ culture, the students contributed to making the school more inclusive by officially acknowledging and welcoming gender diversity by asking all their classmates by which pronoun(s) and the name they wished to be addressed:

*Introducing yourself with your pronouns, yes, I think it’s very important! […] It would be nice to do an introduction sheet with your name, the name you’d like to be called by, it’s safe to be called in class, with your parents … The pronouns you’d like to be used in class… (…) Yeah, it’s starting to become the norm.* [Laughing at people from “old generations” identifying as girls or boys.]

This practice will be introduced at the beginning of each year among middle school students at Lab School Paris.

These results will be discussed along with study 2 results.

## 4. Study 2: Construction of Identity in Adolescence

As mentioned previously, this research was initiated when students started meeting in school to discuss gender and sexual orientation issues that they were confronted with, and that caused discomfort to some of them. With time, the discussion topic enlarged to identity formation, and the research group designed a survey that questioned dimensions of identity that the adolescents perceived as most important. This section presents the results of these questions from the survey.

The survey also included questions that go beyond the scope of the present paper and will be presented in a subsequent article, such as the Consciousness of one’s responsibility scale (Hagège et al. 2021) and an adapted version of the Cantril ladder of satisfaction with life (Levin and Currie 2014).

All co-researcher students identified as LGBTQ+ and were most interested in the topic of gender identity and diversity, although such an interest is growing in society and research (Perry et al. 2019; Rubin et al. 2020).

Gaining a better knowledge of gender identity is particularly important in the case of adolescents who identify as non-binary, a-gender, or genderqueer, as little is known about them (Jones et al. 2016), or their experiences of schooling (Paechter et al. 2021). Most studies about non-binary adolescents focus on social background and mental health and indicate that they are particularly vulnerable, with high rates of depression, anxiety, and suicidal ideation, and risk of experiencing more abuse and victimization than cisgender people (Chew et al. 2020; Jackson et al. 2022; Van der Vaart et al. 2022; Pullen Sansfaçon et al. 2020; Richards et al. 2016). On the other hand, gender self-acceptance (i.e., being satisfied with one’s self-defined gender identity) is negatively associated with stress and positively associated with life satisfaction and perceived academic achievement, which confirms the importance of the recognition of gender diversity and of cultivating gender-identity safe school environments (Day et al. 2018; Watson et al. 2021).

### 4.1. Participants

The participants included 1210 middle school and high school pupils, aged 11 to 18 (M = 15.54, SD = 1.71). Participants’ self-identified gender was female (60.2%), male (32%), non-binary (5.1%), and subjects indecisive about their gender (2.3%). Individuals were described as non-binary when they did not self-categorize as exclusively female or male but as either the combination of the two or as something else, following Galupo et al. (2017) and Hyde et al. (2019). Five subjects chose not to answer the question relative to gender identity and were excluded from further analyses, including the gender identity variable. 

### 4.2. Measures

The online survey assessed several sets of information:

Social and demographic information: The participants indicated their age, living environment (small to medium city/large city), school grade, parents’ occupation, nationality, and religion. The coders defined family socioeconomic status (SES) based on the participants’ description of their parent’s occupations. Then they assigned values to the rank of the occupation type resulting in lower, middle, and upper SES, following the 2020 INSEE categories and through a procedure similar to Lignier and Pagis (2017).

Dimensions important to identity were measured from six questions about how important the following dimensions were about their identity: (1) leisure activities, (2) religion, (3) politics/activism, (4) cultural origin, (5) gender, and (6) sexual orientation. The questions were rated on a 7-point Likert scale from unimportant to very important.

### 4.3. Procedure

The survey was administered online. The first author contacted middle and high schools across France. All the co-researchers also disseminated information using e-mails, newsletters, and social media. Respondents were informed about the research aims and data confidentiality and provided informed consent.

Ethical approval procedures are not yet systematically required in educational science in France for non-interventional studies such as surveys (Claudot et al. 2009). New approval procedures are gradually implemented, but not all institutions have the adequate infrastructure to apply for formal approval before any research (Carvallo 2019). We, therefore, submitted the present research project to two researchers from Swiss institutions (the Haute École pédagogique du Valais and the Centre interfacultaire en Droits de l’enfant, université de Genève) as well as a deontologist/ethic officer from the French Agence de biomédecine, who gave their approval for the study.

### 4.4. Data Analysis

Here we report the analysis of the six dimensions important for identity. How important leisure activities, politics, religion, cultural origin, sexual orientation, and gender are important to identity formation was analyzed as a function of gender, age, living environment, and SES.

These dimensions were analyzed by a 4 × 3 × 4 × 2 repeated measures ANOVA using SPSS statistics (SPSS Inc. Chicago, Illinois, United States of America), with gender identity (male/female/non-binary/indecisive), SES (upper/middle/low), age (11–12/13–14/15–16/17–18) and living environment (small to medium city/large city) as intrasubject factors. Greenhouse-Geisser correction was applied to *p* values associated with multiple degrees of freedom. Paired *t*-tests were used for 2 × 2 comparisons.

### 4.5. Results

Table 1 reports the sociodemographic characteristics of the 1210 participants. Of those who reported their parents’ occupation (97.4%), 47.3% came from upper SES, 32.1% from lower SES, and 18.1% from middle SES. The sample lived in small to medium cities (64.3%) or large cities (35.5%).

All the dimensions of identity did not receive the same ratings of importance, F(5, 5480) = 18.51, *p* < .001. As described below, the effect of dimension interacted with gender, age, living environment, and SES.

**Figure 3:**
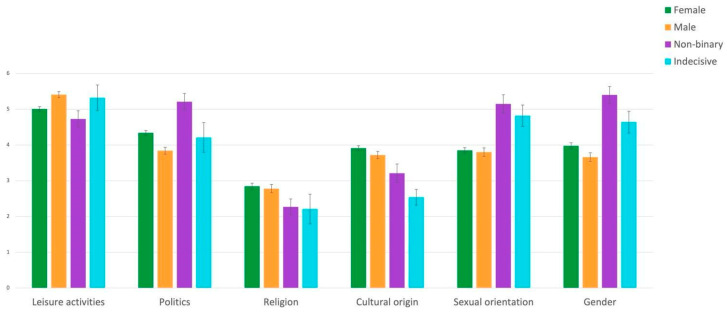
Mean (± SEM) importance for leisure activities, politics, religion, cultural origin, sexual orientation, and gender as a function of gender identity.

Gender identity. Dimension of importance interacted with gender identity, F(15, 5480) = 3.66, *p* < .001. Gender identity had a significant effect on the dimensions of leisure activities (F(3, 1096) = 2.86, *p* = .03), politics (F(3, 1096) = 5.67, *p* < .001), sexual orientation (F(3, 1096) = 7.72, *p* < .001) and gender (F(3, 1096) = 5.67, *p* < .001), whereas there was no effect of gender identity on the dimensions of religion and cultural origin (Figure 3). Post-hoc comparisons showed that the dimensions of sexual orientation and gender were more important to non-binary than male and female subjects (all *p* < .001). Sexual orientation was also more important for indecisive than for female (*p* = .02).

The dimension of politics was more important to non-binary than to male and female (respectively, *p* < .001 and *p* = .003) as well as more important to female than male (*p* = .016). Leisure activities were more important to male than female and non-binary (respectively, *p* < .001 and *p* = .01).

The most important dimensions for indecisive, male and female, were leisure activities (see Table 2 for the associated *p* values), whereas non-binary rated gender, politics, sexual orientation, and leisure activities as the most important dimensions. Religion was the least important dimension for all groups.

Importance interacted significantly with SES (F(10, 5480) = 2.07, *p* = .023). SES impacted the dimension of religion (F(2, 1096) = 3.30, *p* = .03) more importance given to religion in lower SES than upper SES (*p* = .01). There was no effect of SES on any other dimension.

Age interacted with Dimension, F(15, 5480) = 2.12, *p* = .01, with a significant effect of age for sexual orientation (F(3, 1096) = 3.13 *p* = .02) and gender (F(3, 1096) = 3.04, *p* = .028) dimensions only. The youngest group (aged 11–12) rated both sexual orientation and gender less important than the 13–14 years old (respectively, *p* = .01 and *p* = .006), the 15–16 (respectively, *p* = .003 and *p* = .006) and the 17–18 years old (respectively, *p* = .01 and *p* = .04).

Dimension interacted with living environment F(5, 5480) = 2.74, *p* = .028, with the effect of living environment significant for religion (F(1, 1096) = 4.26, *p* = .04) and leisure activities dimensions (F(1, 1096) = 3.93, *p* = .048) only. Religion was more important in large cities than in small to medium cities. Leisure activities were more important in small to medium cities than in large cities.

## 5. Discussion

The current study primarily investigated new ways of fostering the development of 21st-century skills among adolescents through collaborative action research focusing on identity formation. In this section, we will discuss the process of action research, the role of school in fostering identity formation, and skills related to a harmonious and prosperous development into adulthood (study 1). In discussing the various dimensions that adolescents considered most important in defining their identity (study 2), we will focus on the sociodemographic factors related to these dimensions and on elements that are directly relevant to the primary object of our study. We will conclude with the limitations and recommendations for further research.

### 5.1. Looking Back at the Action Research Process

Our research process shared all the characteristic features of action research: immersion of the researchers in the situation; work unfolding in response to a specific situation and not to the researcher’s requirements; questions and problems emerging from the local context; building of descriptions and theoretical frameworks within the context; iteration and tests within the situation; close collaboration between researchers and actors (Holwell 2004). However, it went further than usual action research, classically defined as “a form of self-reflective inquiry undertaken by participants in social situations to improve the rationality and justice of their own practices, their understanding of these practices, and the situations in which the practices are carried out” (Carr and Kemmis 1986, p. 162).

A specific feature of our research is the coexistence of two intertwined levels: the students’ needs that called for intervention and the decision of the educational team to launch a collaborative action research, not only as an answer to those needs but also as a means to reach an even higher target. Indeed, although the project originated from the students’ concerns about gender identity, the implemented setting made it possible to work beyond the initial problem of developing 21st-century skills, as if feeding two birds with one seed.

**Figure 4:**
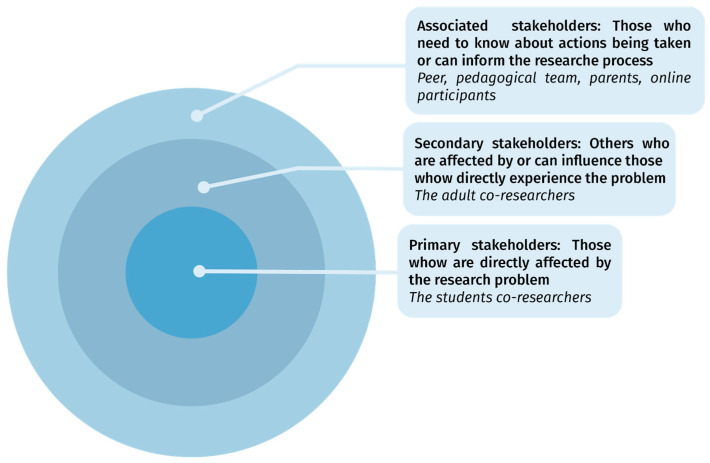
Level of involvement of the participants in the two studies (based on Stringer and Ortiz Aragon’s stakeholders groups model, 2021).

However, the level of implication differed depending on the stakeholders’ statuses. Resorting to the stakeholders groups model proposed by Stringer and Ortiz Aragon (2021), it can be said that the primary stakeholders’ goal—the students—was to get a better understanding of their own identities as adolescents, whereas the secondary stakeholders’ goal—the researchers and the educational team—was to explore new ways of fostering 21st-century skills among adolescents through the research process. As represented in Figure 4, the primary stakeholders’ goal is embedded in the second goal: having a better understanding of adolescents’ identities can indeed help find ways to foster their 21st-century skills.

**Figure 5:**
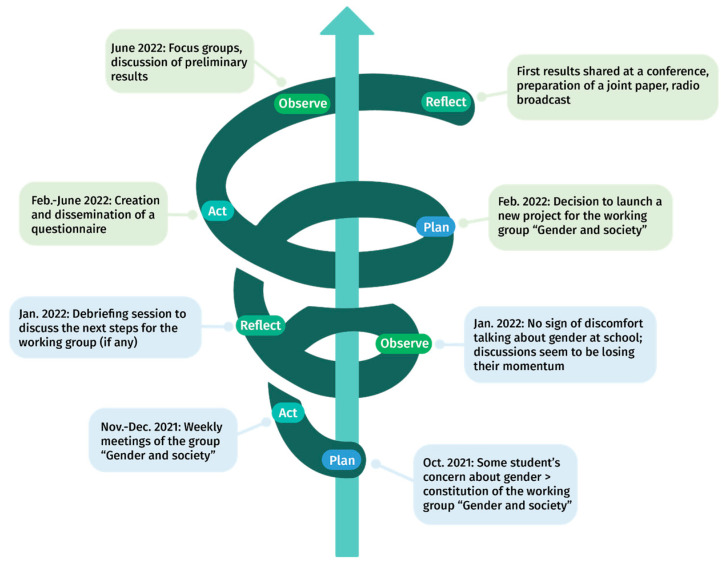
Summary of the activity cycles in our action research, inspired by Kemmis and McTaggert’s cycle (2014).

Action research is a dynamic process involving recurring cycles of activity, sometimes also called self-reflective spiral of cycles—e.g., plan, act, observe, reflect (Kemmis et al. 1988, 2014; Nazari 2021); thinking, planning, doing, and evaluating (Bilorusky 2021)—, and characterized by principles of participation, iteration, inventiveness, and emergence (Burns and McPherson 2017). Figure 5 describes the two cycles that made up our journey: the first one, from October 2021 to January 2022, paved the way for the actual action research cycle, from February to July 2022.

Facilitating identity exploration is one of the roles of the school (Denney 2022; Flum and Kaplan 2012; Roeser et al. 2000). While all the participants were not equally involved in the discussions, each one expressed their appreciation of the framework provided by the project. However, it sometimes became complicated to distinguish clearly between the inputs arising from the initial “Gender and Society” group and what specifically came up from the research process. However, the discussions and the research design provided opportunities to reflect and learn, find significance in their and others’ contributions, and feel more empowered or more aware than before the beginning of the project. Using accurate data to work on real-world problems that concern them personally has successfully fostered motivation and engagement in young people as co-researchers (Jacquez et al. 2020). Throughout the process, we followed Kaplan and colleagues’ four design-based principles to guide teachers in facilitating student identity exploration (2014): (1) promoting personally relevant topics and issues concerning students’ daily lives; (2) triggering identity exploration through personal reflection; (3) maintaining a safe school environment; (4) scaffolding exploratory activities to facilitate students through their identity’s exploration.

All the adults who took part in the process were also aware that they could, as role models, play a critical role in providing templates for young people to develop their identities (Harter 2012; Schwartz and Petrova 2018). Lab School Paris’ pedagogical approach seeks to promote the students’ social and emotional development in various ways (Haag and Martin 2021). This pilot project aimed to support further the students’ self-determined motivation, self-efficacy, and engagement. This is coherent with other studies showing that educational contexts encourage positive civic outcomes, which promote supportive environments for identity exploration while offering critical and analytic awareness of societal issues (Adams and Fitch 1983; Denney 2022; Kaplan et al. 2014; Manganelli et al. 2015). The role of schools as “arenas for exploration and socialization where young people experiment with different roles, values, and relationships” is crucial in the case of “adolescents living in poor and working-class urban communities and deprived of enough opportunities for exploration outside schools” (Abbasi 2016, p. 106).

### 5.2. Gender Identity and Important Dimensions for Identity

The results of study 2 informed on dimensions important for adolescents’ identity, as defined by the adolescent co-researchers themselves: leisure activities, politics/activism, religion, cultural origin, sexual orientation, and gender. The importance of these dimensions varied according to gender identity, SES, and living environment.

Age had little impact on the importance of identity dimensions. However, the 11–12 years old rated sexual orientation and gender as less critical than older age groups, whereas other dimensions did not vary across age. This can be understood because sexual feelings mostly emerge in adolescence, prompting less interest in sexual orientation in late childhood (Diamond and Savin-Williams 2009).

Religion was more important in lower than upper SES and large cities than in small to medium cities. The interpretation of these results calls for caution. How religion is related to development depends on the cultural context, which also depends on various factors such as sociodemographic status and living environments (urban vs. rural) (Good and Willoughby 2007). In further analyses of some variables from our survey that were not considered in the present paper, religiosity should be regarded given its relationship with SES, living environment, and religion (Trommsdorff 2012a). Still, some studies demonstrate considerable variance in adolescents’ religious practices and experiences (Smith and Lundquist-Denton 2005). While religion is assumed to be important in adolescent development, no simple generalizations are possible from the literature results (Trommsdorff 2012b).

Leisure activities were important to identity development (Leverson et al. 2012a), although leisure activities bring together a vast set of activities, some having beneficial or detrimental effects (Freire 2013; Shaw et al. 1995; Stattin et al. 2005). Our questionnaire asked how important leisure activities were to identity, with no possibility of explaining which activity was considered. Male participants rated leisure activities more important than non-binary and female respondents. Previous research has demonstrated substantial differences between male and female in leisure activity choices, with some researchers pointing out that the presentation of leisure activities may be gender stereotypical (Leverson et al. 2012b). Moreover, non-binary young people report barriers in accessing sports practice, resulting in a lower rate of engagement in sports activity (Herrick and Duncan 2018).

The dimension of politics and activism was more important to non-binary than female and male participants, a well-documented effect in the adult non-binary population. For example, more than three-quarters of non-binary adults U.S. citizens reported being registered to vote in 2014 compared to 65% of the U.S. population (James et al. 2016). According to Arnold-Renicker et al. (2020), activism is embraced by non-binary communities to establish their rights and protections. Research has also found an increased interest in political issues among young women in the last 20 years (Briggs 2008), decreasing the gap between male and female. Our results follow this trend, with young women aged 11 to 19 more interested in politics than male participants.

Not only did non-binary participants find the politics and activism dimension more important to identity than male and female participants, but sexual orientation and gender dimensions were also more important to non-binary teenagers than the other groups. Being able to put labels or having words to describe their identity constitutes a turning point for non-binary adolescents (Rankin and Beemyn 2012), who then engage in essential processes of self-reflection and self-education (Bragg et al. 2018). This was evidenced in study one, with co-researchers all identifying as LGBTQ+, but also by study 2 showing that identity dimensions of sexual orientation and gender were significantly more important to non-binary adolescents than other gender groups.

A portion of the participants was indecisive about their gender identity (2.3%). This is not an isolated phenomenon: respondents in an extensive survey of more than 2000 participants, primarily LGBTQ from 15 years old, included about 9% of individuals who did not know how to self-characterize their gender (Richard 2019). Not only can self-categorization be an ongoing process, but its stability can also vary across individuals (Jackson et al. 2022). Self-categorization refers to the capacity to state, describe and articulate one’s gender and includes several processes: an internal sense of gendered self, gendered attributes, other people’s perception, and knowledge of gender in the world (Jackson et al. 2022). Gender self-categorization is a dynamic process across the lifespan.

The results of these two studies offer complementary insights into the question of identity formation in adolescence. They show that identity is determined by different factors that are inextricably connected and the product of both individual characteristics and the context in which they evolve (Lazzeri 2013). They also show how, through the whole research process—especially the construction of the survey and the analysis of the results, some of which are presented in study 2—adolescents became more aware of those various dimensions, thereby getting a better understanding of who they were. Moreover, the discussions allowed them to reflect critically on social norms and explicit or implicit expectations of the various groups to which they belong (family, friends, school, etc.), giving them tools to analyze complex social situations and to become more assertive in those around them. Finally, the research setting also allowed both adults and young co-researchers to experiment with new pedagogical models and build more horizontal and collaborative relationships. Research indicates that identity exploration in school has been associated with motivation, engagement, positive coping, openness to change, flexible cognition, and meaningful learning (Kaplan et al. 2014); in our research, the participants’ attitudes were clearly in line with those observations. It is worth noting that our project took place throughout the school year, allowing a progressive integration of new skills for each participant according to their needs and pace. In the long run, whether this experiment will benefit the participants remains to be investigated.

### 5.3. Limitations

A significant limitation of this study was the limited scope of the research setup: it was prompted by the demand of a group of students with very homogeneous characteristics in the sense that all of them identified as LGBTQ+. They all come from privileged social backgrounds, primarily associated with high levels of cultural capital (e.g., teachers, researchers, company directors); most of them have had the opportunity to live in or visit multiple countries, thus opening up internationally. Three of the five adolescent co-researchers had a natural exposure to scientific research, one of their parents or both working in academia. Likewise, in our sample, parents’ occupations revealed that the upper classes were overrepresented in the collected data. Adapting this setting to a traditional school environment to achieve generalizable results would require substantial adjustments.

Another limitation lies in the short time frame within which the research has been conducted, as pointed out by the participants themselves, which restricted opportunities to work with the adolescent co-researchers on outcomes and dissemination of the project’s result.

### 5.4. Recommendations for Future Research

One way to strengthen our findings will be to replicate this action research in various school contexts to build surveys that reflect the identity-related concerns of more diverse social backgrounds and reach more diverse participants. Improvements suggested by the co-researchers should be considered, such as integrating the whole process into the curricular activities and starting earlier in the school year.

Contrasting with action research in single situations, Holwell (2004) insists on the concept of iterability: to address criticisms made to research-action for its lack of generalization, such methodologies should be possible to adapt to different situations. It may not be feasible to replicate this action research on other sites by bringing in several researchers each time. However, a well-planned and rigorous methodology of co-research, with detailed guidelines for the teachers and student co-researchers, could realistically be implemented under the supervision of a research coordinator in the framework of a collaborative project.

Future research should include a valid assessment of the efficiency of action research with adolescent co-researchers in fostering social and emotional skills and engagement and reflexivity, combining both quantitative and qualitative approaches.

Scholars working on identity and adolescence have pointed out since the late 1990s that identity construction is challenging in our society (Baumeister and Muraven 1996)^2^. Adolescents are vulnerable to risk factors, including emotional, relational, and behavioral problems (Aldam et al. 2019). With adolescents representing a significant proportion of the global population (16% in 2022), understanding and describing identity development during adolescence remains an essential objective for research (Lannegrand-Willems 2012). In the current context, which is particularly anxiogenic due to climate change (Marks et al. 2021; Salomon et al. 2017), special attention must be paid to interventions that can improve young people’s personal resources and skills and build resilience for coping with life’s adversities and challenges (Taylor 2020). Intervention programs should consider the identity processes of exploration and commitment mobilized by individuals in the investigation of the self, relationships with others, and the social world, to accompany and support the various dimensions of identity construction in adolescence (Lannegrand-Willems 2017), which corresponds to what has been broadly defined earlier as 21st-century skills.

Educational teams must carefully monitor the implementation of interventions. Indeed, there is no such thing as ‘one size fits all’ (Pressman and Cross 2018). Even protocols generally considered the most rigorous—large-scale randomized controlled trials—are not always conclusive (Lortie-Forgues and Inglis 2019). Moreover, interventions’ effects can differ substantially depending on some social and environmental characteristics of their targets. For instance, interventions designed to improve psychological health may not only be ineffective but may even produce detrimental effects in some children, notably the most vulnerable ones, such as deterioration in well-being or increased scores on anxiety or depression scales (Das et al. 2016; Montero-Marin et al. 2022). In the specific case of identity, Lannegrand-Willems and Bosma (2006) point out that in a comparison between three high schools, students’ exploration and commitment were higher in the school with students from higher socioeconomic backgrounds. In contrast, Kroger and Marcia (2011) note that differential intervention strategies must be targeted at individuals according to their identity statuses to be efficient. Above all, each child should be supported in a way that respects and fosters their needs and opens a range of possibilities, allowing them to explore various facets of their identity in a secure environment and harmoniously develop both their academic and non-academic skills.

## 6. Conclusions

This school intervention fostered engagement and motivation based on a co-research process with adolescents. It led to more comfort, a better understanding of their identities, and, more generally, identity formation in a group of five adolescents. This action research resulted in a survey administered to 1210 adolescents that informed on dimensions important to identity formation. Mostly gender diversity modulated the relative importance of dimensions to identity formation, pointing out the relevance of educational contexts in promoting a supportive environment for identity exploration.

As our world faces environmental and social problems that current solutions cannot address, there is a growing demand in the field of education to explore new ways to address these increasingly complex challenges. Addressing significant issues for the students and opening up opportunities for them to make their voices heard and take responsibility is beneficial in terms of academic success and the development of their social, emotional, and civic skills. Beyond that, fostering 21st-century skills ultimately aims to enable young people to play their role as active citizens in society fully.

## Figures and Tables

**Table 1:** Sociodemographic characteristics.

	N	%	Missing Values
**Gender Identity**			N = 5
Female	728	60.2	
Male	387	32	
Non-binary	62	5.1	
Indecisive	28	2.3	
**Age**			N = 0
11–12	80	6.6	
13–14	234	19.3	
15–16	388	39.9	
17–18	413	34.1	
**Living Environment**			N = 3 (0.2%)
Small to medium city	778	64.3	
Large city	429	35.5	
**SES**			N = 31 (2.6%)
Lower	388	32.1	
Middle	219	18.1	
Upper	572	47.3	

**Table 2:** *p* values associated with paired *t*-tests resulting from within-dimension comparisons for each gender identity.

Gender Identity	Dimension	Politics	Religion	Cultural Origin	Sexual Orientation	Gender
Female	Leisure activities	<.001	<.001	<.001	<.001	<.001
	Politics		<.001	<.001	<.001	<.001
	Religion			<.001	<.001	<.001
	Cultural origin				0.01	0.20
	Sexual orientation					0.04
Male	Leisure activities	<.001	<.001	<.001	<.001	<.001
	Politics		<.001	0.77	0.058	0.04
	Religion			<.001	<.001	<.001
	Cultural origin				0.112	0.07
	Sexual orientation					0.73
Non-binary	Leisure activities	0.23	<.001	<.001	0.616	0.20
	Politics		<.001	<.001	0.606	0.772
	Religion			0.03	<.001	<.001
	Cultural origin				<.001	<.001
	Sexual orientation					0.21
Indecisive	Leisure activities	0.064	<.001	<.001	0.43	0.46
	Politics		0.004	0.002	0.42	0.42
	Religion			0.87	<.001	<.001
	Cultural origin				<.001	<.001
	Sexual orientation					0.95

## Data Availability

The data that support the findings of this study are available on request from the corresponding author, (P.A.) The data are not publicly available due to the fact that only part of the whole dataset was analyzed for this paper.

## Notes

1 For a short reflexive account of the initial work of the “Gender and society” group, see https://en.labschool.fr/post/philosophical-discussion-as-a-starting-point-for-research (accessed on 15 July 2022).

2 https://www.unfpa.org/data/world-population-dashboard (accessed on 15 July 2022).
